# Assessment of Postoperative Pain, Anxiety, and Functional Recovery Among Patients Undergoing Elective Abdominal Surgery

**DOI:** 10.7759/cureus.85311

**Published:** 2025-06-03

**Authors:** Maheen Zahid, Faisal Wali Ahmed, Fariha Altaf, Misbah Vakil, Sangeetha Sarthi Haleshappa, Rabeea Mazhar, Farhana P Akhter, Asya Batool, Cathrine Nixon, Abdullah Bin Naeem, Saad B Waris

**Affiliations:** 1 Medicine and Surgery, Combined Military Hospital (CMH) Lahore Medical College and Institute of Dentistry, Lahore, PAK; 2 Medicine, King Saud Medical City, Riyadh, SAU; 3 Obstetrics and Gynaecology, Tameside and Glossop Integrated Care NHS Foundation Trust, Manchester, GBR; 4 Medicine and Surgery, Akhtar Saeed Medical and Dental College, Lahore, PAK; 5 Internal Medicine, Nanjing Medical University, Nanjing, CHN; 6 Surgery, Quaid-E-Azam Medical College, Bahawalpur, PAK; 7 Surgery, Lincoln American University, Georgetown, GUY; 8 Surgery, Baqai Medical University, Karachi, PAK; 9 Surgery, Burjeel Medical City, Abu Dhabi, ARE; 10 Orthopaedics, Pakistan Institute of Medical Sciences, Islamabad, PAK; 11 Medicine, Lumina Research Foundation, Islamabad, PAK

**Keywords:** anxiety, elective abdominal surgery, functional recovery, postoperative pain, psychological distress

## Abstract

Introduction: Elective abdominal operations are frequently undertaken to enhance health benefits but tend to result in postoperative recovery delay because of inadequately controlled pain, anxiety, and compromised functional recovery.

Methods: A cross-sectional descriptive study was performed in 300 elective abdominal surgery patients, such as hernia repair, cholecystectomy, and bowel resections. The data were gathered using standardized instruments: the Numeric Rating Scale (for pain), the Hospital Anxiety and Depression Scale (for anxiety), and the Quality of Recovery-15 (for functional recovery). Correlation, t-tests, analysis of variance, and multiple regression analyses were applied. Data gathering was done from December 2024 to April 2025.

Results: Participants reported an average pain score of 4.79 (SD = 1.2), with an anxiety score of 38.4 (SD = 4), while their recovery score was 62 (SD = 16.8). The majority of 184 (61%) participants were men, representing the 26-35 age group among 300 volunteers. Gallbladder removal stood as the most frequently performed operation, with 118 cases (39%) among participants. Pain showed a weak negative correlation with anxiety (r = -0.057, p < 0.001) but a moderate positive correlation with recovery (r = 0.245, p < 0.001), suggesting that higher pain scores were associated with better perceived recovery. Anxiety demonstrated an inverse relationship with recovery based on the statistical results (r = -0.247, p < 0.001). A total of 216 patients (72%) who had undergone previous surgery showed both increased anxiety (M = 38.7, SD = 4.0) and reduced recovery (M = 60.6, SD = 13.8) than patients who had not undergone any surgical procedures (p = 0.007 and p = 0.002). Patients using postoperative pain medication had higher anxiety (M = 38.8, SD = 4.04) and lower recovery scores (M = 59.8, SD = 14.6) than nonusers (p = 0.001 and p < 0.001, respectively), though causality cannot be inferred due to the cross-sectional design. Results from multiple regression indicated that pain input reflected a β value of 0.232 while anxiety demonstrated a β value of -0.233, both predicting recovery at a statistical significance level of p < 0.001.

Conclusion: Both pain and anxiety affect postoperative recovery, but psychological distress impacts more negatively. Holistic postoperative care that considers both physical and psychological health is critical to improve surgical recovery outcomes.

## Introduction

Historically, patients undergoing elective abdominal surgeries face delayed postoperative recovery because healthcare providers are worried about intestinal stoppage, sic, knees, stomatic failures, and loss of appetite. Recent improvements in fast-track rehabilitation programs, combined with the increased effectiveness of pain and nausea management strategies, drive healthcare providers toward encouraging patients to begin consuming foods orally faster for better recovery results [1]. Elective abdominal surgeries present substantial postoperative pulmonary risks that are linked to elevated cardiac risk while affecting patients with multiple medical conditions and showing results of abnormal chest radiographs and abnormal lung examinations. Risk assessment methods require better approaches because preoperative spirometry has proved unable to predict postoperative complications [2].

The prediction of postoperative complications in abdominal cancer surgery depends on the patient's age, preoperative anemia, ASA physical status, and complete fluid management [3]. The risk for death and complications during abdominal surgery remains high among nonagenarians, particularly during emergencies, leading to the demise of half of all patients within 12 months [4].

Patients suffering from sarcopenia experience significantly greater dangers of mortality and complications, along with more extended hospitalization during postoperative recovery for emergencies and elective abdominal surgeries. The risk of death within 30 days after emergency surgical procedures substantially increases when patients have sarcopenia [5]. Postoperative pain management has shown suboptimal results since recent advancements, while medical professionals continue to rely on opioids as a standard treatment. Additional pain management strategies based on regional anesthetics and procedure-specific protocols constitute crucial elements for enhancing treatment success [6]. Patient recovery requires effective postoperative pain relief methods based on multiple therapeutic strategies to reduce opioid medication side effects [7].

The severity of postoperative anxiety is determined by the presence of multiple risks, which include ASA status, smoking history, preoperative anxiety, and postoperative pain [8]. Children experience higher postoperative anxiety risk when they have excessive preoperative anxiety and poor sedation combined with no blocks for pain management and intense postoperative pain [9].

Functional recovery delays with persistent deficits occur in older patients when hospitals need to readmit them after elective surgery. Any two readmissions to the hospital during the initial two months or following a gap between 12 and 18 months strongly raise the chance of persistent functional damage [10]. Studies reveal that older adults reach preoperative functional ability through postoperative recovery within two months after their surgery, with a 65.4% success rate. Research findings demonstrated that patients who did not exhibit aging-related disabilities due to frailty and underwent elective operations tended to recover their functional abilities more effectively [11]. Research reveals that patients undergoing elective surgery experience slow recovery when mobility, pain, and anxiety persist, which negatively affects their health status scores and disability measures during the three-month postoperative period [12].

The purpose of this study is to examine both pain intensity and anxiety symptoms along with functional recovery patterns in patients following elective abdominal procedures. Investigating these variables' relationships can lead to more effective management strategies and better patient-centered postoperative treatment approaches.

Rationale

Healthcare providers conduct elective abdominal surgeries, which serve to help patients achieve better health outcomes and enhance their quality of life. The postoperative recovery period is often hindered by challenges such as pain, anxiety, and impaired functional ability. The insufficient treatment of postoperative pain results in lengthened hospitalizations along with higher complication possibilities, leading to persistent pain issues. Preoperative and postoperative anxiety affects recovery outcomes negatively because it modifies how patients experience pain and controls their general condition. Nonetheless, the speed at which patients achieve functional recovery functions alongside functional ability to establish the total surgical outcome success.

Studies exist that evaluate postoperative pain and anxiety independently, but researchers have not conducted detailed research about how these elements synergistically affect functional recovery. The research fills a knowledge gap by evaluating how pain and anxiety together affect recovery results for patients undergoing scheduled abdominal surgical procedures. Knowledge of this multifactor relationship allows healthcare providers to develop patient-specific postoperative care approaches, which lead to better health results and improved patient happiness.

Objectives

The main research goal evaluates the strength of postoperative pain after elective abdominal surgery while analyzing its relationship to anxiety measures. The study aims to measure anxiety levels of surgical patients after medical procedures as well as investigate their functional recovery in relation to these anxiety levels. The research goal focuses on monitoring patient functional recovery by assessing both their physical comfort and emotional well-being, as well as their capability to handle routine daily tasks. The research examines relationships between intensive pain experiences with anxiety states, plus functional outcome improvement with a specific focus on postoperative variable associations. The study will determine which patient demographic characteristics, such as age and gender, and existing health conditions, contribute to unique outcomes in pain, anxiety, and recovery. The research objectives will provide vital knowledge about postoperative experiences, enabling healthcare providers to develop enhanced care strategies that enhance patient recovery results. We hypothesized that higher levels of postoperative pain and anxiety would be strongly correlated with worse functional recovery outcomes.

## Materials and methods

Study design

The researchers conducted a cross-sectional descriptive study to evaluate postoperative pain management, anxiety levels, and functional recovery in patients who underwent scheduled abdominal surgical procedures. As this was a cross-sectional observational study, no randomization was performed. Several outcome measures were assessed in the postoperative period through evaluation of patient pain intensity, anxiety levels, and recovery status. Using this design, researchers were able to capture a glimpse of patients' surgical recovery process as it stood immediately after their operation.

Study setting

The study took place within a hospital by examining patients who underwent operations of hernia repair or cholecystectomy and bowel resections in surgical wards alongside postanesthesia care units (PACUs). The hospital facility offered extensive surgical and recovery services to create an appropriate research setting.

This research included patients who were 18 years old and above and who underwent scheduled abdominal surgeries while demonstrating their ability to provide consent and complete questionnaires. The research excluded participants who underwent unexpected surgical procedures for their abdomens and individuals with either severe psychiatric diseases or diminished cognitive abilities that prevented them from answering questionnaires. Data collection took place from August 2024 to March 2025.

Sample size and technique

The study used an infinite population formula to calculate the sample size since the overall population was not exactly known. The formula is given as follows:

Sample size = Z² × p(1 - p) / d²

In this, Z is the statistical value at the desired level of confidence, p is the expected proportion or prevalence, and d is the margin of error or precision. For a 95% confidence level, the value of Z is 1.96, and the margin of error is fixed at 0.05. The assumed prevalence was obtained from a similar study in Pakistan with almost the same participants, which had a prevalence of 40.7%. Thus, p is fixed at 0.407. With these values applied, the estimated sample size is 385 [13].

The study team sequentially selected participants throughout the patient admissions for elective abdominal surgery. Participants granted informed consent to researchers before the data collection period, but the researchers asked potential participants who met the inclusion criteria to join. A sufficiently large participant number was chosen to evaluate pain and anxiety management as well as operative function recovery because this number represented the population undergoing similar elective abdominal surgeries.

Data collection tools and procedures

The data were collected using structured standardized questionnaires to ensure uniformity and accuracy of responses among participants. Data collection tools included the Demographic Information Form, the Numeric Rating Scale (NRS) to measure the intensity of postoperative pain, the Hospital Anxiety and Depression Scale (HADS) to assess anxiety levels, and the Quality of Recovery-15 (QoR-15) questionnaire to evaluate functional recovery after surgery. All instruments used were validated and widely considered reliable in clinical research situations. The Demographic Information Form collected basic participant details, including age, gender, marital status, educational level, type of surgery, medical comorbidities, and past surgeries. The NRS is an 11-point scale used by patients to rate the level of their pain, ranging from 0 (no pain) to 10 (the worst imaginable pain). It was developed by Downie et al. in 1978. The reliability of the test-retest has been rated at 0.96 and 0.95, respectively, as per the coefficients of correlation [14]. The 14-item scale measures anxiety and depression in patients present in the hospital. It comprises two seven-item scales: anxiety and depression. All items are scored on a 4-point Likert scale (0-3). The higher the score, the more anxiety and/or depression an individual may experience. It was developed by Zigmond and Snaith in 1983. The HADS-Anxiety subscale yielded a Cronbach's alpha ranging between 0.68 and 0.93 (mean = 0.83), while for the HADS-Depression subscale, it was between 0.67 and 0.90 (mean = 0.82) [15]. The Quality of Recovery-15 is a 15-item survey that focuses on recovery evaluation across multiple levels, including physical comfort, emotional state, physical independence, psychological support, and pain. Each item has been scored from 0 to 10, with a larger total score representing a better recovery outcome. It was developed in 2013 by Stark et al. The internal consistency was high, with Cronbach's alpha showing a score of 0.85 [16].

Once potential participants were identified as meeting the inclusion criteria, they were preoperatively approached for consent. Baseline anxiety levels were assessed, in addition to demographic data, which were recorded preoperatively. Intensity of pain was measured postoperatively with the NRS at around 6, 24, and 48 hours, under standard ward procedure. It was timed according to the feasibility and availability of staff, rather than fixed, hard-set intervals. Anxiety postoperatively was measured with the HADS, usually on postoperative day 2, subject to the patient's state and availability. Functional recovery was evaluated with the QoR-15 on the third postoperative day or at discharge. All surveys were completed in person by a research team member; however, no standardized setting (e.g., a private room) or rigid scheduling protocol was enforced.

Ethical considerations

Data collection was conducted by trained research assistants who provided verbal instructions and assistance when necessary. All information collected was held confidential and kept secure for subsequent analysis. The research study was approved by the Institutional Review Board (IRB) of Lumina Research Foundation, Islamabad, Pakistan (IRB-2024-0093) just before the preparation of initiating research activities.

Data analysis

Data were analyzed using the Statistical Package for the Social Sciences version 26 software (IBM Corp., Armonk, NY). The statistical analyses utilized are descriptive statistics for summarizing demographic information, Pearson correlation to assess relationships among variables, and independent t-tests to compare the two groups' means. One-way analysis of variance (ANOVA) is utilized to compare means in more than two groups, whereas multiple regression tests the effect of predictors on the quality of recovery. Finally, chi-square tests investigate associations among categorical variables such as chronic illness, gender, and length of hospital stay. These analyses serve to investigate differences, relationships, and predictors in the data. The threshold for statistical significance is that the p value is less than 0.05.

## Results

Table 1 shows that a total of 300 participants were studied through a diverse demographic analysis. The 300 participants were predominantly within the age range of 26-35 years (150 people or 50%), yet there were also participants within the age brackets 18-25 (n = 67, 22%) and 36-45 years old (n = 55, 18%), 46-55 years old (n = 20, 7%), and 56-65 years old (n = 8, 3%). Most respondents identified as men (n = 184, 61%) while female respondents made up 29% (n = 87) and 10% (n = 29) chose not to share their gender. Most study participants were married yet remained dispersed among various marital categories, which included single (n = 87, 29%), divorced (n = 66, 22%), widowed (n = 27, 9%), and married (n = 120, 40%).

**Table 1:** Demographic characteristics of participants (n = 300) f: frequency

Variable	f (%)
Age	
18-25 years	67 (22)
26-35 years	150 (50)
36-45 years	55 (18)
46-55 years	20 (7)
56-65 years	8 (3)
Gender	
Male	184 (61)
Female	87 (29)
Prefer not to say	29 (10)
Marital status	
Single	87 (29)
Married	120 (40)
Divorced	66 (22)
Widowed	27 (9)
Educational level	
No formal education	62 (21)
Primary	87 (29)
Secondary	78 (26)
Higher secondary	55 (18)
Bachelors	14 (5)
Masters	4 (1)
Occupation	
Unemployed	77 (26)
Laborer	95 (32)
Professional (e.g., doctor, engineer, and teacher)	69 (23)
Business owner	42 (14)
Retired	17 (6)
Monthly income	
Less than 30,000	93 (31)
30,000-60,000	98 (33)
61,000-100,000	72 (24)
More than 100,000	37 (12)
Type of elective abdominal surgery	
Hernia repair	77 (26)
Gallbladder removal (cholecystectomy)	118 (39)
Appendectomy	77 (26)
Colorectal surgery	28 (9)
Length of hospital stay	
1-2 days	67 (22)
3-5 days	112 (37)
6-10 days	95 (32)
More than 10 days	26 (9)
Previous surgery history	
Yes	216 (72)
No	84 (28)
Chronic illness	
None	75 (25)
Diabetes	105 (35)
Hypertension	66 (22)
Heart disease	31 (10)
Kidney disease	23 (8)
Pain medication use (postsurgery)	
Yes	210 (70)
No	90 (30)
Psychological history	
Yes	216 (72)
No	84 (28)

Participants showed different education levels with primary education (n = 87, 29%) as the most common category while secondary education (n = 78, 26%) followed by no formal education (n = 62, 21%) then higher secondary (n = 55, 18%) and bachelor's degree (n = 14, 5%) and master's degree (n = 4, 1%) being the least common. The research participants consisted mainly of unskilled laborers and unemployed workers (95, 32% and 77, 26%) alongside doctors, engineers, educators, and owners of businesses (69, 23% and 42, 14%). Among the total participants, retired individuals made up 17 cases of the total population (6%), which represented 6%. According to data from participants, most people received wages between Pakistani rupee (PKR) 30,000 and 60,000 (98, 33%) after those earning less than PKR 30,000 (93, 31%) along with those earning between PKR 61,000 and 100,000 (72, 24%) and those earning more than PKR 100,000 (37, 12%). The most frequent procedure among the studied group was gallbladder removal (cholecystectomy) at 118 cases (39%), while hernia repair stood at 77 (26%), appendectomy at 77 (26%), and colorectal surgery at 28 (9%). The hospital stay duration for most patients ranged between three and five days (112; 37%), as well as 6-10 days (95; 32%), with one to two days (67; 22%) and 10 days or longer (26; 9%). A total of 216 doctors (72%) stated they had gone through surgery before, while the remaining 84 (28%) had not. Diabetes emerged as the primary chronic health problem diagnosed among subjects at 105 (35%) compared to hypertension with 66 (22%), heart disease with 31 (10%), and kidney disease with 23 (8%) patients who did not have any persistent health problems at 75 (25%). A total of 210 participants (70%) needed postoperative pain medication, but 90 respondents (30%) did not require it. Moreover, psychological history involved 216 patients (72%), whereas 84 individuals (28%) did not experience it.

Table 2 shows the intercorrelations between study variables: postoperative recovery, psychological distress, and pain intensity. The Numeric Pain Rating Scale was positively and significantly correlated with the Postoperative Quality of Recovery Scale (r = 0.245, p < 0.001), which indicates that more reported pain was related to improved perceived recovery, perhaps indicating early-stage healing or cognitive adjustment to postoperative pain. It also had a high negative correlation with the Hospital Anxiety and Depression Scale (r = -0.057, p < 0.001), although this was weak, suggesting that increased levels of pain were slightly related to lower scores on anxiety and depression.

**Table 2:** Intercorrelations between study variables ^*^p < 0.001 considered significant Pearson's correlation has been used

Variable	Numeric Pain Rating Scale	Hospital Anxiety and Depression Scale	Postoperative Quality of Recovery Scale
Numeric Pain Rating Scale	-	-0.057^*^	0.245^*^
Hospital Anxiety and Depression Scale	-0.057^*^	-	-0.247^*^
Postoperative Quality of Recovery Scale	0.245^*^	-0.247^*^	-

The Hospital Anxiety and Depression Scale was strongly negatively correlated with the Postoperative Quality of Recovery Scale (r = -0.247, p < 0.001), suggesting that higher psychological distress was linked to worse postoperative recovery. All correlations were significant at the 0.001 level, with Pearson's correlation coefficient.

Table 3 presents comparisons between participants with and without a history of prior surgery for pain severity, psychological distress, and postoperative recovery. For the Numeric Pain Rating Scale, no difference was detected between participants with prior surgery (M = 4.73, SD = 1.19) and those without (M = 4.95, SD = 1.22), t(298) = -1.456, p > 0.05, with a small effect size (Cohen's d = -0.18).

**Table 3:** Comparison among variables (previous surgery history) Independent t-test has been used SD: standard deviation

Variable	Yes (n = 216), mean ± SD	No (n = 84), mean ± SD	t	p	95% confidence interval	Cohen’s D
Numeric Pain Rating Scale	4.73 ± 1.19	4.95 ± 1.22	-1.456	>0.05	-0.530 to 0.079	-0.18
Hospital Anxiety and Depression Scale	38.7 ± 4.0	37.3 ± 4.06	2.717	0.007	0.387 to 2.42	0.35
Postoperative Quality of Recovery Scale	60.6 ± 13.8	66.9 ± 20.2	-3.119	0.002	-10.35 to -2.34	-0.40

A notable difference was found in the Hospital Anxiety and Depression Scale in which subjects with a history of previous surgery (M = 38.7, SD = 4.00) registered greater psychological distress compared to the others without (M = 37.3, SD = 4.06), t(298) = 2.717, p = 0.007. The confidence interval (CI = 0.387-2.42) suggests a statistically significant difference that has a small-to-moderate effect size (Cohen's d = 0.35).

About the Postoperative Quality of Recovery Scale, those with a history of surgery (M = 60.6, SD = 13.8) rated postoperative recovery quality significantly lower than those without (M = 66.9, SD = 20.2), t (298) = -3.119, p = 0.002. The 95% confidence interval (-10.35 to -2.34) for this significant difference indicates a small-to-moderate effect size (Cohen's d = -0.40).

Table 4 contrasts patients who used pain medication after surgery with those who did not, on measures of pain severity, psychological distress, and postoperative convalescence. Patients who used pain medication (M = 4.76, SD = 1.20) and those who did not (M = 4.90, SD = 1.22) did not differ significantly in reported pain levels, t (298) = -0.720, p > 0.05, with a trivial effect size (Cohen's d = -0.12). But a large difference was observed in psychological distress, with participants who took pain medication reporting greater anxiety and depression scores (M = 38.8, SD = 4.04) compared to those who did not (M = 37.1, SD = 3.80), t (298) = 3.404, p = 0.001. The 95% confidence interval (0.72-2.70) and a moderate effect size (Cohen's d = 0.43) confirm this finding.

**Table 4:** Comparison among variables (pain medication use postsurgery) Independent t-test has been used SD: standard deviation

Variable	Yes (n = 216), mean ± SD	No (n = 84), mean ± SD	t	p	95% confidence interval	Cohen’s D
Numeric Pain Rating Scale	4.76 ± 1.2	4.9 ± 1.22	-0.720	>0.05	-0.41 to 0.19	-0.12
Hospital Anxiety and Depression Scale	38.8 ± 4.04	37.1 ± 3.8	3.404	0.001	0.72 to 2.70	0.43
Postoperative Quality of Recovery Scale	59.8 ± 14.6	68.4 ± 17.7	-4.412	<0.001	-12.5 to -4.79	-0.55

Regarding recovery from the postoperative period, nonusers of pain medication scored much higher for recovery (M = 68.4, SD = 17.7) than those using pain medication (M = 59.8, SD = 14.6), t (298) = -4.412, p < 0.001, 95% CI = -12.5 to -4.79, and moderate effect size (Cohen's d = -0.55).

Table 5 displays a comparison of the intensity of pain, psychological distress, and postoperative recovery in patients with various chronic illnesses through one-way ANOVA. Numeric Pain Rating Scale scores showed no statistically significant differences between the groups, F(4,295) = 0.705, η² = 0.01, suggesting similar pain irrespective of the type of chronic illness.

**Table 5:** Comparison of variables (chronic illness) One-way ANOVA has been used SD: standard deviation; F: F ratio; η^2^: effect size; ANOVA: analysis of variance

Variable	None (n = 75), mean ± SD	Diabetes (n = 105), mean ± SD	Hypertension (n = 66), mean ± SD	Heart disease (n = 31), mean ± SD	Kidney disease (n = 23), mean ± SD	F (4,295)	η^2^
Numeric Pain Rating Scale	4.88 ± 1.19	4.76 ± 1.2	4.63 ± 1.3	5.03 ± 1.2	4.74 ± 1.14	0.705	0.01
Hospital Anxiety and Depression Scale	39.1 ± 3.8	39.2 ± 3.7	37.8 ± 4.2	36.2 ± 4.3	36.3 ± 3.8	5.997	0.08
Postoperative Quality of Recovery Scale	59.4 ± 13.5	58.9 ± 13.6	66.0 ± 18.6	73.4 ± 15.6	62.4 ± 18.7	6.820	0.09

A statistically significant difference, however, was found in psychological distress as indicated by the Hospital Anxiety and Depression Scale, F(4,295) = 5.997, η² = 0.08. Individuals with diabetes (M = 39.2, SD = 3.7) and without illness (M = 39.1, SD = 3.8) exhibited greater distress compared to individuals with heart disease (M = 36.2, SD = 4.3) or kidney disease (M = 36.3, SD = 3.8), indicating chronic illness type influences psychological outcomes.

There was also a large difference in postoperative quality of recovery, F(4,295) = 6.820, η² = 0.09, with the best recovery scores in the heart disease group (M = 73.4, SD = 15.6), then the hypertension group (M = 66.0, SD = 18.6), and the worst in the diabetes group (M = 58.9, SD = 13.6). This indicates that the type of chronic illness is meaningfully linked to differences in recovery after surgery.

Table 6 illustrates the variation in pain severity, psychological distress, and postoperative recovery by gender identity according to one-way ANOVA. There was no statistically significant difference in the Numeric Pain Rating Scale scores between the groups, F(2,297) = 1.154, η² = 0.008, indicating comparable levels of perceived pain for men (n = 184, M = 4.79), women (n = 87, M = 4.68), and those who did not want to report gender (n = 29, M = 5.07).

**Table 6:** Comparison of variables (gender) One-way ANOVA has been used SD: standard deviation; F: F ratio; η^2^: effect size; ANOVA: analysis of variance

Variable	Male (n = 184), mean ± SD	Female (n = 87), mean ± SD	Prefer not to say (n = 29), mean ± SD	F (2,297)	η^2^
Numeric Pain Rating Scale	4.79 ± 1.26	4.68 ± 1.17	5.07 ± 0.96	1.154	0.008
Hospital Anxiety and Depression Scale	38.8 ± 3.90	37.3 ± 3.67	37.9 ± 5.51	4.258	0.03
Postoperative Quality of Recovery Scale	59.9 ± 13.9	66.6 ± 18.5	65.7 ± 18.2	6.076	0.04

Nonetheless, scores for the Hospital Anxiety and Depression Scale were significantly different statistically, F(2,297) = 4.258, η² = 0.03, and the men claimed the highest level of psychological distress (M = 38.8) compared to women (M = 37.3) and the "prefer not to say" category (M = 37.9), suggesting gender is influential in emotional health after surgery.

Likewise, there was a large difference in the Postoperative Quality of Recovery Scale scores, F(2,297) = 6.076, η² = 0.04. Women (M = 66.6) and those who did not want to report gender (M = 65.7) reported improved recovery compared to men (M = 59.9), indicating a possible gender-related effect on recovery perceptions or experiences.

**Figure 1:**
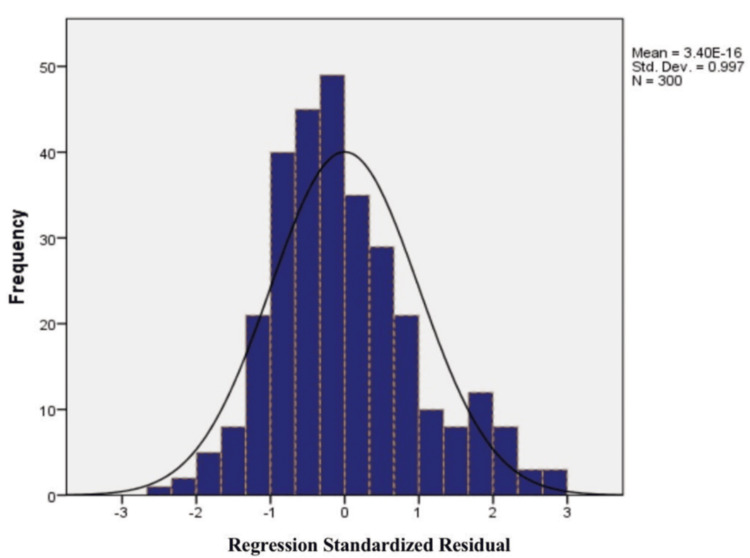
Histogram for multiple regression of postoperative quality of recovery scale

Table 7 shows the outcome of a multiple regression analysis of scores on the Postoperative Quality of Recovery Scale as predicted by two predictors: the Numeric Pain Rating Scale and Hospital Anxiety and Depression Scale. The intercept (constant) of the model was significant (B = 82.93, p < 0.001), which reflects the expected baseline score on the recovery metric when both predictors equal zero. The Numeric Pain Rating Scale was a significant positive predictor of recovery postoperatively (B = 3.085, β = 0.232, p < 0.001), and this implies that for any one-unit increase in the self-report pain, the recovery score is increased by roughly 3.09 units, although this finding might be due to reverse coding or reading of the recovery scale. On the other hand, the Hospital Anxiety and Depression Scale was a strong negative predictor (B = -0.922, β = -0.233, p < 0.001), which shows that increased anxiety and depression levels are linked with reduced postoperative recovery quality. Both predictors made significant contributions to variance in postoperative recovery outcomes, highlighting the significance of controlling both physical and psychological aspects following surgery. Figure 1 presents the histogram for the regression standardized residuals for the Postoperative Quality of Recovery Scale. The residuals are distributed around zero, with a mean approximately equal to 0 and a standard deviation of 0.997, verifying a good fit for the model. Furthermore, the near-normal distribution supports the assumptions of regression and indicates that the model results can be trusted.

**Table 7:** Multiple regression for postoperative quality of recovery scale Constant: Postoperative Quality of Recovery Scale ^*^p < 0.01 considered significant B: coefficient; SE: standard error; β: standardized coefficient

Variable	B	95% confidence interval	SE	β	p
Constant	82.93	64.8 to 101.1	9.211	-	<0.001^*^
Numeric Pain Rating Scale	3.085	1.65 to 4.52	0.727	0.232	<0.001^*^
Hospital Anxiety and Depression Scale	-.922	-1.35 to -0.497	0.216	-0.233	<0.001^*^

Table 8 shows the prevalence of chronic disease by gender and duration of hospital stay among postoperative patients. Of the total sample, 75 (20.8%) had no chronic disease, 105 (29.2%) had diabetes, 66 (18.4%) had hypertension, 31 (8.6%) had heart disease, and 23 (6.4%) had kidney disease. During the analysis of gender differences, among the controls, 51 were men (68%), 18 were women (24%), and six did not wish to disclose (8%). Of the participants with diabetes, 68 participants were men (64.8%), 29 participants were women (27.6%), and eight participants (7.6%) did not wish to disclose their gender. Similar patterns continued for other types of chronic illnesses, with an overrepresentation in male participants. A chi-square test indicated a statistically significant gender-chronic illness association (χ² = 7.87, p = 0.05), which implies that gender distribution significantly differs across various categories of chronic illnesses.

**Table 8:** Descriptive statistics of demographic variables (chronic illness, gender, and length of hospital stay) p indicates the level of significance; p values are calculated using the chi-square test; the significance level is set at p < 0.05 f: frequency

Variables (chronic illness)	f	Gender		Prefer not to say	p	x^2^	One to two days	Three to five days	Length of hospital stay 6-10 days	More than 10 days	p	x^2^
		Male	Female									
None	75	51	18	6	0.05	7.87	13	34	19	9	0.35	13.23
Diabetes	105	68	29	8			28	36	36	5		
Hypertension	66	33	26	7			14	25	22	5		
Heart disease	31	18	8	5			7	8	10	6		
Kidney disease	23	14	6	3			5	9	8	1		

As for the length of stay at the hospital, patients with no chronic disease spent their time mainly in three to five days (n = 34, 45.3%) or six to ten days (n = 19, 25.3%). Among diabetes patients, most spent their time mainly at three to five days (n = 36, 34.3%) or six to ten days (n = 36, 34.3%). Likewise, patients with hypertension, heart disease, and kidney disease had differential distribution between one to two, three to five, six to ten, and over ten days. Yet, the chi-square test revealed no statistically significant correlation between chronic illness and hospital stay duration (χ² = 13.23, p = 0.35), indicating that the nature of chronic illness did not significantly influence the duration of patients' hospital stays following surgery. Generally, the findings highlight that gender has a major impact on the occurrence of chronic diseases among surgical patients, while hospital stay duration seems to be unrelated to particular chronic diseases.

Table 9 shows the distribution of various types of elective abdominal operations by gender and length of hospital stay. The most common operation was removal of the gallbladder (n = 118; 32.9%), followed by hernia repair and appendectomy (each n = 77; 21.5%), and colorectal surgery (n = 28; 7.8%). There was a statistically significant relationship between the type of operation and gender (χ² = 11.11, p = 0.05). In particular, cholecystectomy was most prevalent among men (n = 78; 66.1%), whereas appendectomy involved a significantly higher number of subjects who did not wish to state their gender (n = 13; 16.9%).

**Table 9:** Descriptive statistics of demographic variables (type of elective abdominal surgery, gender, and length of hospital stay) p indicates the level of significance; p values are calculated using the chi-square test; the significance level is set at p < 0.05 f: frequency

Variables (type of elective abdominal surgery)	f	Gender		Prefer not to say	p	x^2^	One to two days	Three to five days	Length of hospital stay 6-10 days	More than 10 days	p	x^2^
		Male	Female									
Hernia repair	77	46	28	3	0.05	11.11	13	35	19	10	0.05	16.38
Gallbladder removal (cholecystectomy)	118	78	31	9			35	42	36	5		
Appendectomy	77	45	19	13			15	28	27	7		
Colorectal surgery	28	15	9	4			4	7	13	4		

In terms of hospitalization, there was also a significant relationship found (χ² = 16.38, p = 0.05). Those who had gallbladder and appendectomy often stayed for three to five days (n = 42 and n = 28, respectively), while the others with colorectal surgery tended to have a relatively longer stay, with more in the 6-10 day and over 10-day groups (n = 13 and n = 4, respectively). This implies variability in the length of stay according to surgical intervention.

## Discussion

The current research sought to evaluate postoperative pain, anxiety, and functional recovery in patients who underwent elective abdominal surgery. Our research identified a positive relationship between pain severity and perceived recovery, which contradicts another study that revealed increased pain levels were associated with extended recovery periods, indicating the importance of balanced pain management strategies [17]. One explanation could be reverse causality, whereby patients who recover more quickly are likely to be more active and, therefore, suffer more pain from increased mobility. Another possibility is psychological adaptation, where patients interpret moderate pain as a sign of good healing progress [18]. Although our study revealed a poor negative correlation between pain severity and psychological distress, indicating little inverse relationship, another study reported that more severe pain and longer pain duration were associated with a poorer outcome of depression and anxiety. This difference could be due to variations in design, population, or measurement of symptoms over time [19]. Our findings indicated that higher psychological distress was associated with worse recovery, and pain had weak correlations with both distress and recovery. These same trends were seen in past studies, where anxiety and depression were found to have negative effects on surgical recovery, while positive psychological characteristics promoted improved healing [20].

In our research, prior surgical experience did not have a significant impact on current pain severity, although it was associated with increased psychological distress and worse recovery. This contrasts with previous findings that indicated past pain shapes expectations of future pain, and it may indicate a gap between expected and experienced pain [21]. The results of this study corroborate earlier studies that emphasize the presurgery distress in predicting postoperative outcomes. Consistent with these findings, our study revealed that psychological distress before surgery and some expectations affected postoperative recovery, further emphasizing the need to address emotional well-being before surgery [22].

Our findings indicate that the frequency and timing of analgesic administration might not greatly affect pain intensity, as both groups had similar pain scores. This is consistent with evidence that delayed analgesic administration might not be as effective in controlling pain and enhancing recovery [18]. Our results concur with previous literature, emphasizing that the prevalence of chronic conditions and medication use is strongly associated with psychological distress and fatigue [23]. Our findings are consistent with the increasing implementation of improved recovery pathways (ERPs) emphasizing multimodal pain management, demonstrating that decreased opioid use can enhance postoperative recovery outcomes. This is consistent with recent trends supporting nonopioid treatments in pain management [24].

Our results suggest that the nature of chronic illness has a strong impact on psychological distress and recovery after surgery. This concurs with existing research that indicates that sociodemographic determinants like low income, homelessness, and restricted access to primary care are associated with increased chronic illness and pain [25].

Our results showed few gender differences in the severity of pain, with men having slightly higher scores than women. Females did, however, have improved postoperative recovery outcomes, consistent with the current literature that presents women as having superior recovery following surgery. These results support the acknowledgment of gender in postoperative care and recovery planning [26].

Our results agree with the earlier studies, which indicated that preoperative psychological distress is associated with poorer postoperative pain outcomes. The variations in pain and function scores between distressed and nondistressed patients were small and indicate that the effect of psychological distress might not be clinically relevant at the individual level [27]. The results showed a substantial link between gender and chronic illness occurrences since men demonstrated higher prevalence rates across diverse medical conditions. These results support previous research, which validates differences in illness behavior and psychological responses between genders after surgery because these factors affect recovery and treatment adherence [28]. Our research discovered no substantial relationship between chronic health conditions and patients' hospital stay period. Previous studies have shown that total comorbidity scores are superior to age in predicting hospital stay duration [29]. The results showed that the nature of performed abdominal surgeries directly affected the presence of male and female patients as well as the time they spent in the hospital. Research findings validate prior work that demonstrates gender-specific surgical outcomes due to variables such as surgical complexity and patient age, which primarily impact male patients [30].

Limitations and future research

While this study provides valuable insights into the relationships between pain, anxiety, and recovery, several limitations should be acknowledged. The study employed a cross-sectional design, which means that causality cannot be firmly established. Future longitudinal studies are needed to track the long-term effects of pain and anxiety on recovery outcomes. Additionally, the study population was limited to patients undergoing elective abdominal surgery, and the findings may not be generalizable to other surgical populations. Future research could explore these variables in different types of surgeries and diverse patient demographics.

Another limitation is the reliance on self-reported measures for both pain and anxiety. Although these tools are commonly used in clinical research, they are subject to individual biases and may not fully capture the complexity of these variables. Objective measurements, such as physiological markers of anxiety or pain levels, could offer additional insights into the mechanisms underlying postoperative recovery. Also, although the research compared results among patients and controls who were users or nonusers of postoperative pain medication, it did not distinguish between medication types (e.g., acetaminophen, NSAIDs, and opioids). This makes it difficult to assess the efficacy of analgesic regimens. Future studies should stratify by medication type to identify what works best to enhance postoperative recovery.

## Conclusions

The current research emphasizes the powerful effects that pain, together with anxiety, has on postoperative recovery following elective abdominal surgery. The research data show that pain intensity and emotional distress produce negative recovery results, but patients experience better outcomes from proper pain treatments with local anesthetics and ketorolac administration. The treatment of postoperative anxiety leads to better functional recovery, together with reduced recovery duration. Patient recovery requires complete strategies that unite pain control and emotional care, so recovery becomes optimal and clinical results are enhanced.

## References

[1] Bisgaard T, Kehlet H (2002). Early oral feeding after elective abdominal surgery---what are the issues?. Nutrition.

[2] Lawrence VA, Dhanda R, Hilsenbeck SG, Page CP (1996). Risk of pulmonary complications after elective abdominal surgery. Chest.

[3] Simões CM, Carmona MJ, Hajjar LA (2018). Predictors of major complications after elective abdominal surgery in cancer patients. BMC Anesthesiol.

[4] Racz J, Dubois L, Katchky A, Wall W (2012). Elective and emergency abdominal surgery in patients 90 years of age or older. Can J Surg.

[5] Hajibandeh S, Hajibandeh S, Jarvis R, Bhogal T, Dalmia S (2019). Meta-analysis of the effect of sarcopenia in predicting postoperative mortality in emergency and elective abdominal surgery. Surgeon.

[6] Rawal N (2016). Current issues in postoperative pain management. Eur J Anaesthesiol.

[7] Garimella V, Cellini C (2013). Postoperative pain control. Clin Colon Rectal Surg.

[8] Caumo W, Schmidt AP, Schneider CN (2001). Risk factors for postoperative anxiety in adults. Anaesthesia.

[9] Caumo W, Broenstrub JC, Fialho L (2000). Risk factors for postoperative anxiety in children. Acta Anaesthesiol Scand.

[10] Pisani MA, Albuquerque A, Marcantonio ER (2017). Association between hospital readmission and acute and sustained delays in functional recovery during 18 months after elective surgery: the successful aging after elective surgery study. J Am Geriatr Soc.

[11] Becher RD, Murphy TE, Gahbauer EA, Leo-Summers L, Stabenau HF, Gill TM (2020). Factors associated with functional recovery among older survivors of major surgery. Ann Surg.

[12] Ferraz SM, Moreira JP, Mendes LC, Amaral TM, Andrade AR, Santos AR, Abelha FJ (2018). Evaluation of the quality of recovery and the postoperative health status after elective surgery. [Article in Portuguese]. Braz J Anesthesiol.

[13] Naing L, Nordin RB, Abdul Rahman H, Naing YT (2022). Sample size calculation for prevalence studies using Scalex and ScalaR calculators. BMC Med Res Methodol.

[14] Downie WW, Leatham PA, Rhind VM, Wright V, Branco JA, Anderson JA (1978). Studies with pain rating scales. Ann Rheum Dis.

[15] Zigmond AS, Snaith RP (1983). The hospital anxiety and depression scale. Acta Psychiatr Scand.

[16] Stark PA, Myles PS, Burke JA (2013). Development and psychometric evaluation of a postoperative quality of recovery score: the QoR-15. Anesthesiology.

[17] Pavlin DJ, Chen C, Penaloza DA (2002). Pain as a factor complicating recovery and discharge after ambulatory surgery. Anesth Analg.

[18] Montgomery GH, Bovbjerg DH (2004). Presurgery distress and specific response expectancies predict postsurgery outcomes in surgery patients confronting breast cancer. Health Psychol.

[19] Gerrits MM, Vogelzangs N, van Oppen P, van Marwijk HW, van der Horst H, Penninx BW (2012). Impact of pain on the course of depressive and anxiety disorders. Pain.

[20] Mavros MN, Athanasiou S, Gkegkes ID, Polyzos KA, Peppas G, Falagas ME (2011). Do psychological variables affect early surgical recovery?. PLoS One.

[21] Walmsley PNH, Brockopp DY, Brockopp GW (1992). The role of prior pain experience and expectations on postoperative pain. J Pain Symptom Manage.

[22] Melzack R, Abbott FV, Zackon W, Mulder DS, William M, Davis L (1987). Pain on a surgical ward: a survey of the duration and intensity of pain and the effectiveness of medication. Pain.

[23] Koopmans GT, Lamers LM (2000). Chronic conditions, psychological distress and the use of psychoactive medications. J Psychosom Res.

[24] Cheung CK, Adeola JO, Beutler SS, Urman RD (2022). Postoperative pain management in enhanced recovery pathways. J Pain Res.

[25] Hanley O, Miner J, Rockswold E, Biros M (2011). The relationship between chronic illness, chronic pain, and socioeconomic factors in the ED. Am J Emerg Med.

[26] Vallerand AH, Polomano RC (2000). The relationship of gender to pain. Pain Manag Nurs.

[27] Crisson JE, Keefe FJ (1988). The relationship of locus of control to pain coping strategies and psychological distress in chronic pain patients. Pain.

[28] Modica M, Ferratini M, Spezzaferri R, De Maria R, Previtali E, Castiglioni P (2014). Gender differences in illness behavior after cardiac surgery. J Cardiopulm Rehabil Prev.

[29] Rochon PA, Katz JN, Morrow LA, McGlinchey-Berroth R, Ahlquist MM, Sarkarati M, Minaker KL (1996). Comorbid illness is associated with survival and length of hospital stay in patients with chronic disability. A prospective comparison of three comorbidity indices. Med Care.

[30] Harten J, McCreath BJ, McMillan DC (2005). The effect of gender on postoperative mortality after emergency abdominal surgery. Gend Med.

